# Assessment of toxic heavy metals in commonly consumed foods in Egypt and their implications for public health and safety

**DOI:** 10.1038/s41598-025-27798-w

**Published:** 2025-12-03

**Authors:** Khaled Salahel din, Yasmine Abdalbasit, Abdelbaset Abbady, Nagwa Saad

**Affiliations:** 1 Physics Department, Faculty of Science, South Valley University, Qena, 83523 Egypt; 2 Faculty of Computer and Information, South Valley University, Qena, 83523 Egypt

**Keywords:** Heavy metals, Foods and beverages, Health risk, Egypt, Environmental sciences, Risk factors

## Abstract

The current study provides a comprehensive analysis of toxic heavy metal concentrations, specifically lead (Pb), cadmium (Cd), chromium (Cr), and arsenic (As), in various commonly consumed food categories in Egypt, including beverages, processed cereals, milk/dairy products, canned fish/meat products, and table salt. The results indicate that Pb levels were the highest among the metals analyzed, with the highest level found in canned fish/meat products (average: 221.5 ± 39.9 µg/kg). Cd, Cr, and As were present in lower amounts, also with the highest levels detected in canned fish/meat products (averaging 45.8 ± 52.8, 36.2 ± 41.3, and 8.5 ± 1.8 µg/kg, respectively), which raises concerns regarding dietary exposure. Dietary exposures were quantified using estimated daily intake (EDI), with values reaching up to 0.688 µg/kg body weight/day for Pb, 0.112 µg/kg/day for Cd, 0.035 µg/kg/day for Cr, and 0.004 µg/kg/day for As. Health risks were evaluated using hazard quotients (HQ) and carcinogenic risks (CR) in accordance with guidelines from the United States Environmental Protection Agency (US EPA). All HQ values were below 1, indicating no significant non-carcinogenic health risk, while CR values ranged from 2.16 × 10⁻⁹ to 4.37 × 10⁻⁵, which are within the US EPA’s acceptable lifetime cancer risk range of 10⁻⁶ to 10⁻⁴. Specifically, an HQ of less than 1 suggests no significant health concern, and CR values within this range indicate acceptable cancer risk levels. Pb consumption may lead to a minor increase in systolic blood pressure (0.53 mmHg), while urinary cadmium levels in adults (1.06 µg/g creatinine) were found to be safe. Although these results suggest no significant health risks from heavy metal consumption for the local population, ongoing monitoring and regulatory actions are essential to mitigate potential health risks associated with heavy metal exposure through food.

## Introduction

Food plays a vital role in human nutrition, providing proteins, vitamins, and minerals, but it can also contain harmful pollutants such as heavy metals (H.Ms), which are dense elements (> 5 g/cm³). Contamination with H.Ms is a global issue, threatening food safety and human health. While essential metals like Iron (Fe), Zinc (Zn), Copper (Cu), and Manganese (Mn) are beneficial in small amounts, nonessential metals like Lead (Pb), Arsenic (As), Mercury (Hg), Cadmium (Cd), and Chromium (Cr) are toxic even at low exposure levels^1^. H.Ms originate from both natural sources “earth’s crust” and human activities such as mining, industry, and agriculture. Excessive use of metallo-pesticides, phosphate fertilizers, and irrigation with contaminated water has increased their presence in the environment^2^. Due to their non-biodegradable nature, they accumulate in soil and water for several decades owing to their high rate of soil-to-plant transfer and then to livestock, eventually entering the food chain^3,4^. Commonly consumed foods such as cereals, meat, fish, milk, etc. can contain traces of these metals, making ingestion the primary route of human exposure^5^. Long-term consumption of contaminated foods leads to the bioaccumulation of H.Ms in vital organs such as he kidney, liver, bone, and central nervous system, resulting in severe health consequences such as immunosuppression, biochemical disruptions, neuromuscular defects, and carcinogenic effects^6,7^. Pb, Cd, Cr, and As are highly toxic, lack biological function, and are identified as priority food pollutants by the U.S. Environmental Protection Agency (USEPA) and European Food Safety Authority (EFSA), unlike essential metals, which have a lower toxicity threshold^1^. Arsenic (As), a toxic metal found in various ecosystems, is a potent carcinogen. Epidemiological evidence^8,9^ indicates that long-term exposure to inorganic arsenic in drinking water at or above ~ 50 µg/L is associated with significantly elevated risks of bladder and kidney cancers. Lead (Pb), a widely used metal, is a potential carcinogen causing severe health issues. It harms children’s development and cognition, while adults may suffer hypertension and neurological damage^10^. According to epidemiological data, even low levels of dietary Pb exposure are linked to subtle but measurable rises in adults’ systolic blood pressure (roughly 0.5–1.0.5.0 mmHg for every 1–2 µg/dL increase in blood lead)^11–14^ Cadmium (Cd), a toxic metal released into the environment mainly from industrial processes, is strongly linked to renal dysfunction and other long-term health effects, with studies showing that adult renal impairment occurs at urinary cadmium (UCd) levels above 5 µg/g creatinine, a threshold that correlates with biomarkers of kidney injury and corresponds to approximately 100 mg/kg Cd in the renal cortex^15–17^. While Chromium (Cr), particularly in its hexavalent form (Cr(VI)), is a toxic carcinogen associated with lung cancer and skin disorders^18^. Given the growing concerns over H.Ms contamination in food, assessing their levels in widely consumed food products in Egypt, which may be contaminated by industrial activities and agriculture, is crucial. However, health risk assessments on foodborne H.Ms remain limited. The prior studies in Egypt have mostly focused on a limited number of food items^19–22^; highlighting the need for a comprehensive assessment, particularly of less-studied foods like soft drinks and instant noodles. This study specifically targets (Pb), (Cd), (Cr), and (As) because these metals are considered top priority toxicants for food safety on both global and Egyptian scales. The United States Environmental Protection Agency (USEPA) and the International Agency for Research on Cancer (IARC) classify them as hazardous substances due to their carcinogenic, neurotoxic, and systemic toxic effects. Moreover, previous research has highlighted their occurrence in Egyptian agricultural and food products, emphasizing their importance in regional health risk assessments. This focus ensures that the study addresses the metals that pose the greatest concern for human health risks associated with dietary exposure in Egypt. Currently, no unified national standards exist in Egypt for H.Ms in food; therefore, this study used international guidelines (WHO/FAO, EFSA, and USEPA) as references. This regulatory gap highlights the need for baseline data to inform future national limits. Accordingly, the present study aims to measure the concentration of toxic heavy metals (Pb, Cd, Cr, and As) in five commonly consumed food categories in Egypt, such as beverages, processed cereals, milk/dairy products, canned fish/meat products, and table salt, and assess their potential health risks for adult consumers.

## Materials and methods

### Collection and digestion of samples

Fifty-four food and beverage samples were collected between January and December 2022 from randomly selected local markets in Qena Governorate, South of Egypt, to represent commonly consumed daily items. The samples were categorized into five groups: beverages (20 samples), processed cereals (18 samples), milk/dairy products (6 samples), fish/meat products (6 samples), and table salt (4 samples).

Samples were collected in test tubes without any additional treatment. Fish/meat products were first cut into small pieces and mixed in a grinder until homogeneous dough was formed. Liquid, dairy, and fish/meat product samples were stored in the refrigerator until the time of analysis.

Foodstuff samples (0.5 mL liquid and/or 0.5 g solid) were mixed with 10 mL of concentrated (98%) sulfuric acid (H_2_SO_4_) in a digestion flask and left overnight. The mixture was then evaporated on a hot plate, adding (98%) perchloric acid (HCIO_4_) until all organic matter was destroyed and the sample turned white. After cooling to room temperature, the solution was filtered and diluted to 50 mL with deionized water and kept at room temperature for H.Ms analysis. Blank samples were prepared using deionized water instead of the bio-sample and digested in the manner as foodstuff samples using metallic acids; their values were subtracted from the studied samples to correct background interference. Calibration standard solutions of Pb, Cd, Cr, and As were prepared by diluting certified standard solutions (1000 mg/L, 99.9%) to the desired concentrations, establishing calibration curves for each element by plotting standard concentrations (4 points) versus their absorbance. A recovery rate of 85–96% was acquired and considered satisfactory for analysis, and method precision was further confirmed through replicate analysis (*n* = 3), yielding RSD values below 10%.

### Sample analysis

Heavy metals Pb, Cd, Cr, and As concentrations in foodstuff samples were measured using Atomic Absorption Spectroscopy (AAnalyst 400, PerkinElmer, USA) at the National Research Centre, Cairo. This analytical technique quantifies metallic concentrations by measuring light absorption at specific wavelengths after sample vaporization, comparing results with a calibration curve. The limits of detection (LOD) and quantification (LOQ) (µg/kg) for Pb were 0.1–0.5 and 0.3–1.5, for Cd 0.05–0.1 and 0.15–0.3, for Cr 0.2–0.5 and 0.6–1.5, and for As 0.1–0.5 and 0.3–1.5.

### Health risk assessment of heavy metals in foodstuffs

The assessment of health risks from exposure to H.Ms through food intake in this study was based on the following parameters:

**The estimated daily intake (EDI)** of H.Ms through foods is the basic variable of health risk assessment. EDI is calculated using the following Eq. 1^23^:

1 $$\:\text{E}\text{D}\text{I}\:=\:\frac{\text{C}\:\times\:\:\text{I}\text{R}\:}{\text{B}\text{W}\:}$$

where C (mg/L) or (mg/kg) is the mean concentration of metal in foods (see Table 1), IR (kg/day/person) is the ingestion rate “average daily consumption among Egyptian individuals of food product (Table 3)”, BW is the average adult body weight (70 kg)^22^. In the absence of Egyptian demographic data, the ingestion rates utilized were derived from national dietary consumption statistics that represent typical food intake among Egyptian adults. An average adult body weight (BW) of 70 kg was selected based on international exposure assessment guidelines to provide more accurate risk estimations. However, it is recognized that the estimated daily intake (EDI) values reflect average exposures for adults and may not adequately account for variations in more vulnerable groups, such as children, pregnant women, or those with differing eating habits.

**Table 1:** Average concentrations ± standard deviation (SD), range, and permissible value of H.Ms in foodstuff groups.

Category	Sample type	Sample number	Concentration (µg/kg)												
			Pb		Cd		Cr		As		Pb		Cd	Cr	As
			Average ± SD	Range	Average ± SD	Range	Average ± SD	Range	Average ± SD	Range	*Maximum permissible limits				
Beverages	Carbonated soft drinks	12	18.7 ± 9.7	8–41	9.3 ± 8.5	1–30	7.7 ± 5.8	2–21	1.5 ± 0.8	ND − 6	**10**		**10**	**50**	**10**
	Energy drinks	2	21.5 ± 5.5	16–27	8.5 ± 4.5	4–13	11 ± 5	6–16	1.6 ± 1.0	ND − 3					
	Tea	3	33.0 ± 9.4	21–44	15.0 ± 3.7	10–19	8.7 ± 0.5	8–9	5.0 ± 0.8	4–6	**500**		**100**	**NA**	**NA**
	Instant coffee	3	14.0 ± 4.1	9–19	8.0 ± 0.8	7–9	5.3 ± 0.5	5–6	2.7 ± 1.2	1–4	**NA**		**NA**	**NA**	**NA**
	**All beverages**	**20**	**20.4 ± 10.3**	**8–44**	**9.9 ± 7.2**	**1–30**	**7.8 ± 5.0**	**2–21**	**4.1 ± 1.4**	**ND-6**					
Processed Cereals	Wheat Flour	5	68.0 ± 21.3	34–96	6 ± 2	3–9	6.8 ± 0.8	5–9	4.0 ± 1.7	1–6	**200**		**100**	**200**	**10–100**
	Bread	3	43.7 ± 16.3	24–64	5.7 ± 1.7	4–8	2.7 ± 1.2	1–4	3.3 ± 1.2	2–5					
	Macaroni	2	45 ± 5	40–50	5.5 ± 1.5	4–7	3.0 ± 0.7	3–3	2 ± 1	1–3					
	Instant noodles	5	97.0 ± 21.4	70–124	7.0 ± 1.4	5–9	8.2 ± 0.7	7–9	6.8 ± 1.6	5–9					
	Corn products	3	24.3 ± 6.8	19–34	7.3 ± 1.7	5–9	4.0 ± 0.8	3–5	3.7 ± 1.2	6–9					
	**All cereals**	**18**	**62.2 ± 31.4**	**19–124**	**6.4 ± 1.8**	**3–9**	**5.6 ± 2.4**	**1–9**	**5 ± 2.4**	**1–9**					
Milk/Dairy milk products	Milk	4	20.8 ± 6.6	12–30	6.3 ± 2.8	3–9	5.3 ± 1.9	3–8	1.8 ± 0.4	1–2	**20**		**10**	**150**	**10**
	Dairy milk	2	25 ± 15	10–40	6.5 ± 1.5	5–8	5.5 ± 0.5	5–6	4.0 ± 0.5	4					
	**All milk**	**6**	**22.2 ± 10.4**	**10–40**	**6.3 ± 2.4**	**3–9**	**5.3 ± 1.6**	**3–8**	**2.5 ± 1.1**	**1–4**					
Fish/Meat products	Canned Fish	2	206 ± 28	178–234	9.5 ± 1.5	8–11	7.5 ± 1.5	6–9	8 ± 1	7–9	**300**		**50**	**100**	**100**
	Canned Beef	2	194 ± 4	190–198	8.5 ± 0.5	8–9	6.5 ± 0.5	6–7	7 ± 1	6–8	**100**		**50**	**100**	**10**
	Luncheon	2	264.5 ± 33.5	231–298	119.5 ± 14.5	105–134	94.5 ± 4.5	90–99	10.5 ± 1.5	9–12					
	**All fish/meat**	**6**	**221.5 ± 39.9**	**190–298**	**45.8 ± 52.8**	**8–134**	**36.2 ± 41.3**	**6–99**	**8.5 ± 1.8**	**6–12**					
Table salt	Processed & natural	4	174.0 ± 38.5	111–211	8.3 ± 1.5	6–10	10.0 ± 4.1	7–17	7.8 ± 0.8	7–9	**2000**		**500**	**NA**	**100**
Total value			3685 (72%)		659 (12.9%)		546 (10.7%)		228 (4.5%)						
Overall heavy metals content			5118												

*H.M permissible limit in foods (EFSA)^15,34,35,51^.

**Hazard Quotient (HQ)** was adopted to assess the potential non-cancer risk of dietary exposure to H.Ms^23^. HQ calculation formula is as follows in Eq. 2^24^:

2 $$\:\text{H}\text{Q}\:=\:\frac{\text{C}\:\times\:\:\text{I}\text{R}\:\times\:\:\text{E}\text{D}\:\times\:\:\text{E}\text{F}}{\text{B}\text{W}\:\times\:\:\text{R}\text{f}\text{D}\:\times\:\:\text{A}\text{T}}$$

where ED (year) is exposure duration (70 years) equivalent to the average lifetime, EF is the exposure frequency (365 days/years), AT (day) is the mean exposure time for noncarcinogens (365 days/year × ED)^25^, RfD is the reference dose (mg/kg/day), which represents the maximum acceptable oral intake dose of a toxic metal without discernible risk over a lifetime^24^, RfD for Pb, Cd, Cr, and As 0.0035, 0.001, 0.003 and 0.0003 mg/kg/day, respectively^26^. The RfD values for Pb, Cd, Cr, and As used in this study were obtained from authoritative toxicological databases, primarily the United States Environmental Protection Agency (USEPA) Integrated Risk Information System (IRIS) and scientific opinions from the European Food Safety Authority (EFSA). These values are specifically applicable to oral/dietary exposure routes, ensuring relevance and reliability for risk assessment of foodborne contaminants.

Cumulative dietary risks from multiple heavy metals were assessed using the Hazard Index (HI=$\:\sum\:\text{H}\text{Q}$), assuming additive effects^1^. HI reflects cumulative exposure this approach assumes additive toxicity and does not capture potential synergistic or antagonistic interactions among metals. The complexity of such interactions and lack of sufficient toxicological data limit precise synergy assessment in dietary contexts.

**Carcinogenic Risk (CR)** cancer risk associated with H.Ms exposure. It presents an upper-limit probability for cancer development in exposed individuals. USEPA sets a permissible lifetime risk range of (1 in 1,000,000) to (1 in 10,000): CR < E-06 means no risk, E-06 < CR < E-04 is acceptable, and CR > E-04 exceeds tolerance^27^. CR calculation formula is as follows Eq. 3^24^:

3 $$\:{C}{R}\:=\:\frac{{C}\:\times{I}{R}\:\times{E}{D}\:\times{E}{F}\:\times{C}{S}{F}}{{B}{W}\:\times{A}{T}}$$

where CSF cancer Slope Factor (mg/kg/day) is used to estimate the potential cancer risks from oral exposure to H.Ms and other carcinogens by quantifying their relative potency in causing cancer, CSF for Pb, Cd, Cr, and As 0.0085, 0.38, 0.5 and 1.5 mg/kg/day, respectively^28,29^. Exposure parameters—including exposure frequency (EF = 365 days/year), exposure duration (ED = 70 years), and averaging time (AT)—follow established USEPA guidelines for lifetime exposure. While ideally these parameters would be tailored to Egypt-specific demographic and epidemiological data, such data remain limited at present. This limitation justifies the use of these conservative default values to ensure a protective assessment. Due to data limitations, uncertainty was not quantified, such as confidence intervals or probabilistic risk assessments.

#### Potential blood pressure effects

Exposure to heavy metals, especially Pb, presents health risks, notably by raising systolic blood pressure. The Joint FAO/WHO Expert Committee on Food Additives (JECFA) states that ingestion of 1.3 µg/kg b.w. per day of Pb can increase systolic blood pressure by 1 mmHg (0.1333 kPa)^30^. Based on this statement the potential increase in blood pressure for Egyptian adults due to Pb intake through foods consumption was calculated.

#### Urinary cadmium (UCd)

To assess the potential risk of renal injury due to Cd intake, the concentration of cadmium in urine (UCd) (µg/g creatinine) for adults was estimated using the following formula Eq. 4^31^.

4 $$\:\text{C}\text{U}\text{r}\text{i}\text{n}\text{e}\:=\:\frac{\text{f}\text{k}\times\:\text{f}\text{u}\:}{\text{L}\text{o}\text{g}\left(2\right)}\times\:\text{E}\text{D}\text{I}\times\:{\text{t}}_{1/2}\times\:\frac{[1-\:\text{E}\text{x}\text{p}\left(-\:\frac{\text{log}\left(2\right)\:\times\:\:\text{a}\text{g}\text{e}}{{\text{t}}_{1/2}}\right)]}{\left[\right(1-\:\text{Exp}\left(-\:\frac{\text{l}\text{o}\text{d}\left(2\right)}{{\text{t}}_{1/2}}\right)]}$$

where EDI: is the average daily intake of Cd in the diet (µg/kg b.w. day), t_1/2_ : Biological half-life of Cd (12.7 years), (f_u_ :Fraction of Cd absorbed and f_k_ :Fraction of Cd excreted) f_k_×f_u_ refers to the absorption rate of the gastrointestinal tract and renal cortex (0.0076); age: Age of the individual is set at 50-year-olds according to the European Food Safety Authority (EFSA)^16^. The safety limit for UCd (5.24 µg/g creatinine) was set by JECFA^30^. The impact of Pb on systolic blood pressure was estimated based on JECFA guidance. Similarly, Cd-related renal risks were assessed by estimating UCd concentrations using established pharmacokinetic models. These associations with biomarkers are based on published reference studies. It is recognized that direct validation through biomonitoring data from the Egyptian population would strengthen the exposure-response relationship, and this recommendation is presented as a recommendation for future work.

## Results and discussion

### Heavy metals concentration in foodstuffs

**Table 1** presents the concentrations of toxic heavy metals Pb, Cd, Cr, and As (µg/kg) found in different samples of foods and beverages commonly consumed in Egypt. The results indicate substantial variability in heavy metal levels across the analyzed items. Pb exhibited the highest concentrations, with values ranging from 8 µg/kg in carbonated drinks to 298 µg/kg in luncheon. Cd levels varied from 1 µg/kg in carbonated drinks to 134 µg/kg in luncheon. Cr concentrations were between 1 µg/kg in bread and 99 µg/kg in luncheon. As showed the lowest levels, ranging from undetectable (in 71% of carbonated and energy drinks) to 12 µg/kg in luncheon.

The overall heavy metals content indicates that Pb is the most prevalent, comprising 72% of the total detected heavy metals, followed by Cd (12.9%), Cr (10.7%), and As (4.5%) (Table 1). This distribution underscores the need for targeted interventions to reduce Pb exposure, particularly in high-risk food items. Pb in foods and beverages can originate from environmental contamination (water and agricultural soil), metallic impurities in the added ingredients and preservatives, or contaminated processing equipment and packaging, especially if Pb-coated containers deteriorate and allow Pb to seep into products^32^. Classified as a Group 2B carcinogen in humans, Pb raises significant health concerns^33^. The study found that Pb levels exceeding (10 µg/kg) EFSA limits in 79% of carbonated and energy drink samples, (20 µg/kg) in 50% of milk and dairy products samples, and (100 µg/kg) in 100% of canned beef and luncheon samples^34^. Cd was above permissible limits (10 µg/kg) in 29% of carbonated and energy drink samples and (50 µg/kg) in 100% of luncheon samples^15^, while As exceeded limits (10 µg/kg) in 50% of luncheon samples^35^. The H.Ms levels in the other foodstuffs groups remain within safe limits.

To identify significant differences, one-way ANOVA and Tukey post-hoc tests were performed to analyze the differences in Pb, Cd, Cr, and As concentrations across the studied food groups. The one-way ANOVA results indicate highly significant differences in the concentrations of Pb, Cd, Cr, and As across the five studied food groups (p-values all < 0.0001). Subsequent Tukey HSD post-hoc tests reveal that fish/meat products and table salt consistently exhibit significantly higher concentrations of toxic heavy metals compared to beverages, processed cereals, and milk/dairy products.

Specifically, Pb concentrations in fish/meat products and table salt are significantly elevated, pointing to these food groups as major dietary contributors to Pb exposure in the local population. Cd levels are markedly higher in fish/meat products than all other groups, raising concern for potential renal and other health effects associated with chronic exposure. Similarly, Cr and As concentrations are higher in fish/meat and table salt, underscoring the need for targeted monitoring and control measures in these food categories.

The statistically significant variability in metal concentrations among food groups justifies differential risk assessment approaches and tailored intervention policies to mitigate health risks. Regular surveillance leveraging biomonitoring data is also recommended to validate exposure and affect relationships.

Compared to previous studies in Table 2, the levels of Cd and As in the studied beverages exceeded those found in USA soft drinks^36^, while the levels of Pb and Cd were elevated compared to Polish energy drinks^37^ and Pakistani tea^38^ but were lower than those in Jordanian soft drinks^39^,, Chinese tea^25^, and Peruvian coffee^40^. In processed cereals, wheat flour exhibited higher Pb levels than in China^1^. However, H.Ms levels were lower than those in Jordanian wheat flour and bread^39^, Egyptian macaroni in a previous study^41^, South Korean instant noodles^5^, and Chinese corn^42^. In milk and dairy products, Pb and Cd levels in milk surpassed those reported in France^43^. Cd levels exceeding those in a previous Egyptian study for milk, while dairy products had lower H.M. levels^44^. In fish/meat products, canned fish had higher Pb levels than its Italian equivalent^45^ but less than in Iran^46^. Canned meat presented higher Cd and As levels than in Poland^23^, and luncheon meat had higher Cd levels than in prior Egyptian studies^21^ and Jourden^39^. Finally, Cr and As levels in table salt were elevated compared to Jordan^47^, whereas Pb and Cd levels were lower than those in previous studies from Egypt and Iran^48,49^. Overall, previous Egyptian studies indicated significantly elevated Pb levels, consistent with the results of the current study, suggesting potential environmental pollution.

**Table 2:** Comparison of the H.Ms levels in foodstuffs with similar studies.

Category	Country	Type of drink	Mean concentration (µg/kg)				Reference
			**Pb**	**Cd**	**Cr**	**As**	
Beverages	Egypt	Carbonated soft drinks	18.7 ± 9.7	9.3 ± 8.5	7.7 ± 5.8	1.5 ± 0.8	Present study
		Energy drinks	21.5 ± 5.5	8.5 ± 4.5	11 ± 5	1.6 ± 1	
		tea	33 ± 9.4	15 ± 3.7	8.7 ± 0.5	5 ± 0.8	
		Instant coffee	14 ± 4.1	8 ± 0.8	5.3 ± 0.5	2.7 ± 1.2	
	Jordan	Soft drinks	80–710	10–100	NA	NA	^39^
	USA		NA	0.03 ± 0.08	NA	0.85 ± 0.3	^36^
	Nigeria		46–109	27–160	57–122	17–330	^52^
	Poland	Energy drinks	10.37 ± 8.5	0.34 ± 0.18	39.7 ± 18.3	7.33 ± 7.0	^37^
	Nigeria		12–323	769–779	NA	NA	^53^
	China	Tea	1090	140	1170	210	^25^
	Pakistan		2.1	1.34	NA	NA	^38^
	Iraq		1690 ± 130	110 ± 4	600 ± 20	23 ± 5	^54^
	Poland	Coffee	11–82.6	0.030–3.2	NA	NA	^55^
	Peru		640–730	NA	50–390	500–940	^40^
Processed cereals	Egypt	Wheat Flour	68 ± 21.3	6 ± 2	6.8 ± 0.8	4 ± 1.7	Present study
		Bread	43.7 ± 16.3	5.7 ± 1.7	2.7 ± 1.2	3.3 ± 1.2	
		Macaroni	45 ± 5	5.5 ± 1.5	3 ± 0.7	2 ± 1	
		Instant noodles	97 ± 21.4	7 ± 1.4	8.2 ± 0.7	6.8 ± 1.6	
		Corn	24.3 ± 6.8	7.3 ± 1.7	4 ± 0.8	3.7 ± 1.2	
		Macaroni	245–299	127–155	NA	NA	^41^
	China	Wheat flour	45	23	234	24	^1^
	Jordan		80 ± 30	NA	NA	NA	^39^
		Bread	50–90	NA	NA	NA	
	Ethiopia		1550–3410	180–1650	NA	NA	^56^
	South Korea	Instant noodles	634–820	ND − 714	NA	55–86	^5^
			ND − 3163	ND	ND − 2421	ND	^57^
	China	Corn	210	NA	120	NA	^42^
	Brazil		95	40	NA	47	^58^
Milk and dairy products	Egypt	Milk	20.8 ± 6.6	6.3 ± 2.8	5.3 ± 1.9	1.8 ± 0.4	Present study
		Dairy products	25 ± 15	6.5 ± 1.5	5.5 ± 0.5	4 ± 0.5	
		Milk	45.06	4.77	NA	NA	^44^
	France		9	0.34	NA	NA	^43^
	Peru		29 ± 22	7 ± 6	NA	10 ± 4	^59^
	Palestine		200–930	36–54	NA	NA	^60^
	Egypt	Dairy products	418–745	31.3–51.9	NA	NA	^44^
	France		47	1.37	NA	NA	^43^
Fish and meat products	Egypt	Canned fish	206 ± 28	9.5 ± 1.5	7.5 ± 1.5	8 ± 1	Present study
		Canned meat	194 ± 4	8.5 ± 0.5	6.5 ± 0.5	7 ± 1	
		luncheon	264.5 ± 33.5	119.5 ± 14.5	94.5 ± 4.5	10.5 ± 1.5	
		Canned fish	2030	653	63	NA	^61^
	Italy		159 ± 115	12 ± 16	NA	NA	^45^
	Iran		20–5500	ND − 270	NA	NA	^46^
	Jordan	luncheon	ND − 300	ND − 100	NA	NA	^39^
	Egypt		224–330	53–57	NA	NA	^21^
	Poland	canned meat	202	5	244	2	^23^
Table salt	Egypt	processed and natural	174 ± 38.5	8.3 ± 1.5	10 ± 4.1	7.8 ± 0.8	Present study
		natural	500–1640	10–30	NA	NA	^48^
	Iran	processed	309–379	17–20	NA	NA	^49^
	Jordan		NA	NA	4.74 ± 0.6	4.95 ± 0.8	^47^

N.D: not detected, NA: not applicable.

**Fig. 1:**
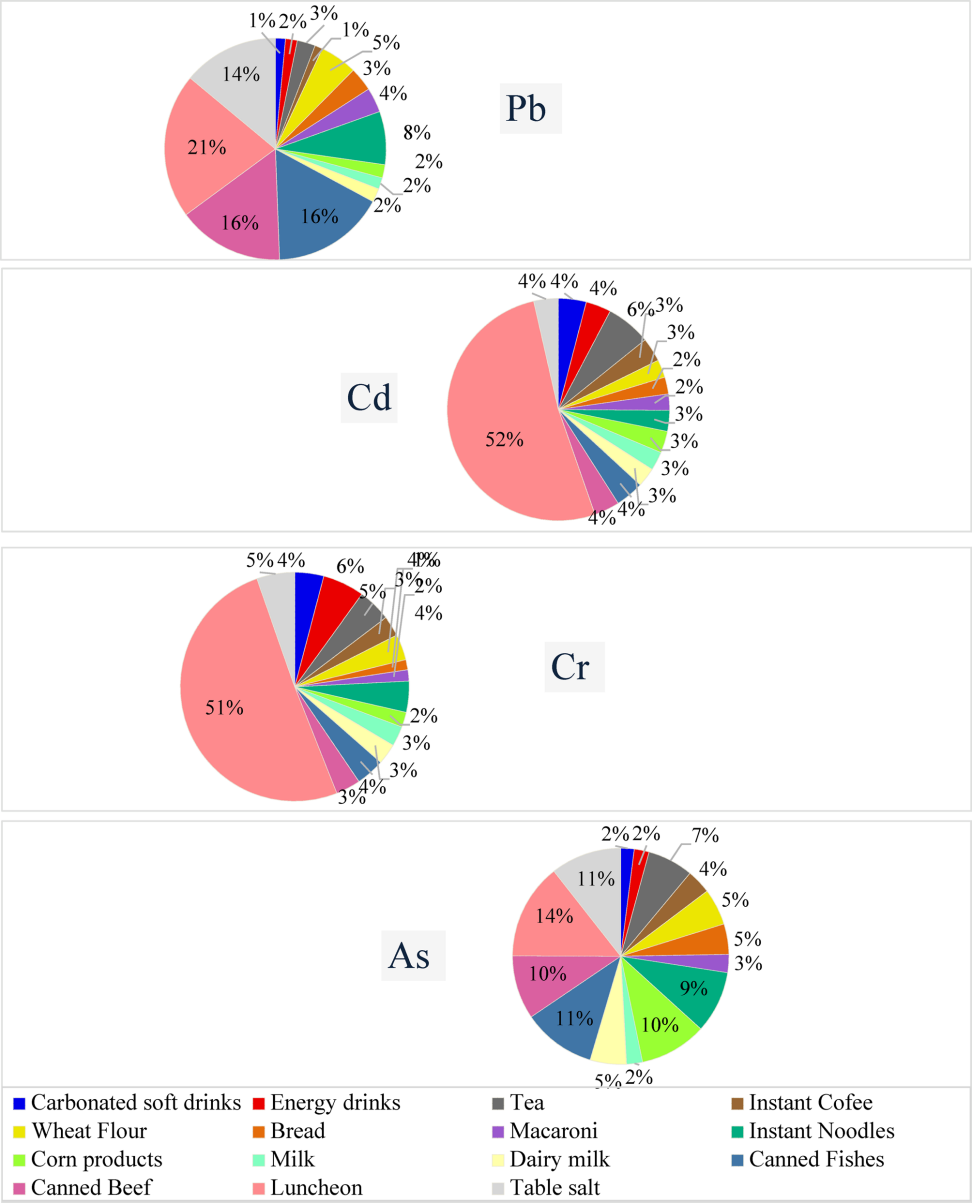
Food groups contributions to studied H.Ms.

The average H.Ms content in studied foodstuff categories ranked as: (fish/meat products > table salt > processed cereals > beverages > milk/dairy products). Figure 1 shows the contributions of various food types to H.Ms analyzed. Luncheon meat was the primary contributor to all H.Ms found in the foodstuffs, accounting for 21% of Pb, 54% of Cd, 52% of Cr, and 15% of As, followed by canned fish (17%) and canned meat (16%) for Pb, tea (7%) for Cd, energy drinks (6%) for Cr, and salt (11%) and canned fish (11%) for As. The remaining food groups had varying contributions to H.Ms. Elevated levels of H.Ms in fish/meat products may result from animals’ bodies, particularly fish, naturally accumulating metals in their fatty tissues through consuming plants and organisms exposed to contaminated environments^39^. Continuous monitoring and broader geographic sampling are recommended to better characterize these patterns. Variabilities in food contamination, consumption habits, and toxicological reference values contribute to overall uncertainty. Incorporating probabilistic methods and sensitivity analyses in future work is encouraged to increase confidence in risk assessment outcomes.

### Health risk of heavy metals

To assess health risks from H.Ms in food consumed by adults, the estimated daily intake (EDI) was calculated and compared to health-based guidance values (HBGVs) from the European Food Safety Authority (EFSA), (Table 3). Pb has a benchmark dose level of 0.63 µg/kg b.w. per day, Cd has a tolerable weekly intake of 2.5 µg/kg b.w. (approximately 0.36 µg/kg b.w. per day), and the tolerable daily intake for (Cr) is 0.3 µg/kg b.w. per day. No safe level exists for (As), so exposure should be minimized as much as possible. Table 3 ranks H.M.‘s EDI as: Pb > Cd > Cr > As, with Pb being the highest contributor due to its significant levels in food samples. Processed cereals, particularly wheat flour, were the main contributors to dietary H.Ms exposure, accounting for 67.1% of the total intake (6.37E-01 µg/kg body weight/day), as they are the main food source for Egyptians. Fish/meat products account for 12.2% (1.73E-01 µg/kg b.w./day), largely due to the high H.Ms content in luncheon samples, while beverages and milk/dairy products contribute 10.8% (1.03E-01 µg/kg b.w./day) and 3.6% (3.45E-02 µg/kg b.w./day), respectively. Table salt contributes minimally at 0.002% (2.04E-03 µg/kg b.w./day). All EDI values are within HBGVs, indicating no health concerns for the population.

**Table 3:** Average daily consumption per individual in Egypt (kg or L/day/person) © Statista 2023 and average estimated dietary intake of H.Ms via food consumption in comparison to allowed values.

Category	Sample type	IR (kg/day or L/day)	Estimated daily intake EDI (µg/kg b.w. per day)				
			Pb	Cd	Cr	As	Total Intake
Beverages	Carbonated soft drinks	0.083	2.23E-02	1.11E-02	9.14E-03	1.76E-03*	4.43E-02
	Energy drinks		2.56E-02	1.01E-02	1.31E-02	1.97E-03*	5.08E-02
	Tea	0.008	3.67E-03	1.67E-03	9.87E-04	6.00E-04	6.92E-03
	Instant coffee	0.002	4.00E-04	2.33E-04	1.50E-04	7.00E-05	8.54E-04
Processed Cereals	Wheat Flour	0.340	3.30E-01	2.91E-02	3.30E-02	1.94E-02	4.12E-01
	Bread	0.099	6.20E-02	8.05E-03	3.79E-03	4.73E-03	7.86E-02
	Macaroni	0.022	1.44E-02	1.76E-03	9.58E-04	6.39E-04	1.77E-02
	Instant Noodles	0.036	4.92E-02	3.55E-03	4.16E-03	3.45E-03	6.04E-02
	Corn products	0.113	3.92E-02	1.18E-02	6.44E-03	1.18E-02	6.92E-02
Milk/Dairy products	Milk	0.063	1.86E-02	5.61E-03	4.72E-03	1.57E-03	3.05E-02
	Dairy milk (cheese & yogurt)	0.004	1.98E-03	8.43E-04	6.67E-04	4.63E-04	3.95E-03
		0.012					
Fish/Meat products	Canned Fishes	0.008	2.35E-02	1.08E-03	8.54E-04	9.11E-04	2.63E-02
	Canned Beef	0.015	4.04E-02	1.77E-03	1.35E-03	1.46E-03	4.50E-02
	Luncheon		5.51E-02	2.49E-02	1.97E-02	2.19E-03	1.02E-01
Table salt	processed & natural	0.0007	1.77E-03	8.40E-05	1.02E-04	7.89E-05	2.04E-03
**Total sample**			6.88E-01	1.12E-01	9.91E-02	5.11E-02	9.50E-01
**Ratio of EDI to HBGVs (%)**			0.06–52.4%	0.02–8.1%	0.03–11%	-	
**Health-based guidance values			**0.63**	**0.36**	**0.3**	**NA**	

*The LOQ value of As was applied when undetected in the sample.

**Health-based guidance values (HBGVs) of H.Ms in food^15,34,35,51^.

**Fig. 2:**
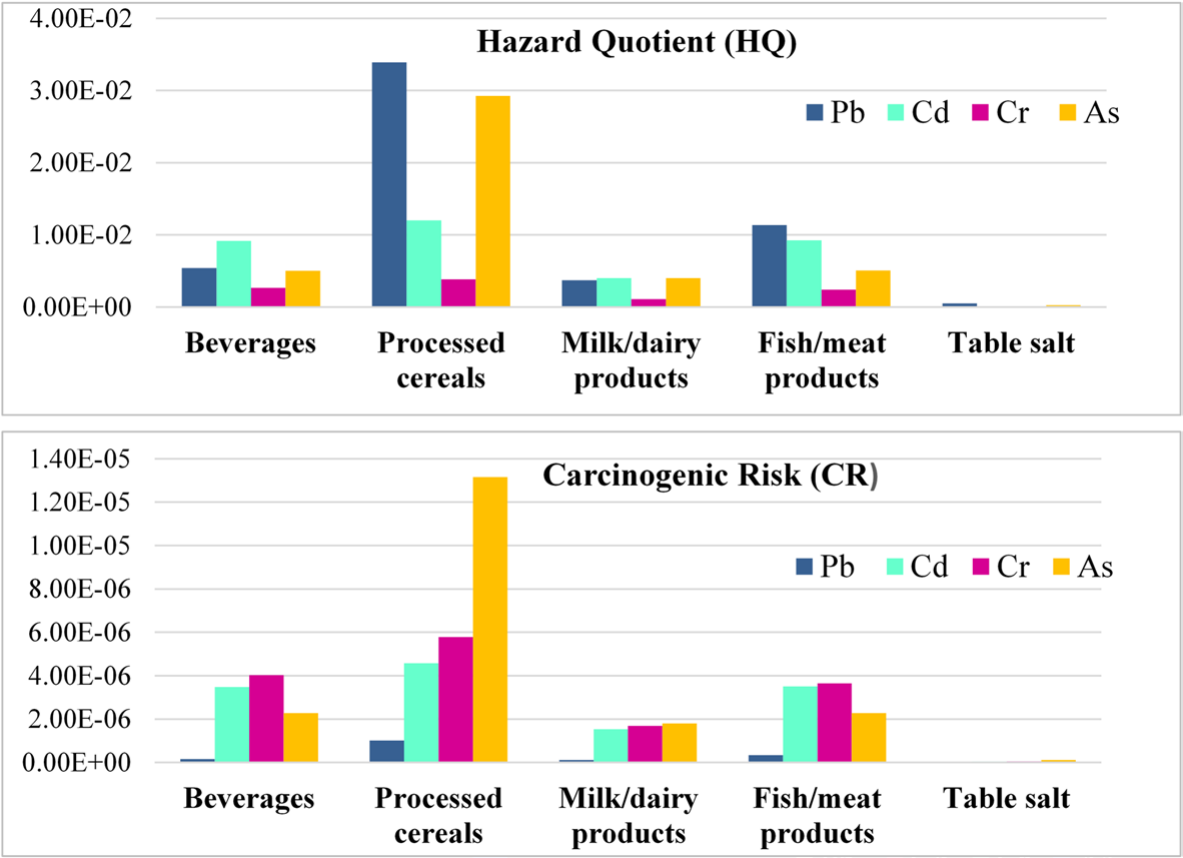
Average (HQ) and (CR) of H.Ms from consumption foodstuff categories.

The Hazard Quotient (HQ) and carcinogenic risks (CR) of heavy metals in the analyzed food samples are presented in Table 4; Fig. 2. HQ values for H.Ms ranged as follows: Pb (7.25E-05 to 1.33E-01), Cd (6.11E-05 to 4.37E-02), Cr (2.37E-05 to 1.46E-02) and As (9.39E-05 to 9.71E-02). All HQ and total HQ (HI) values were below 1, indicating no significant health risks from H.M. exposure^50^. Pb had the highest HQ due to its concentration, contributing 38.9% to the total HQ, followed by As (32.7%), Cd (21.8%), and Cr (6.6%). The CR values were as follows: Pb (2.16E-09 to 3.96E-06), Cd (2.32E-08 to 1.66E-05), Cr (3.56E-08 to 2.18E-05), and As (4.23E-08 to 4.37E-05), all within acceptable cancer risk limits (E-06 < CR < E-04) asserting no health concerns from the studied H.Ms^28^. The relative contributions to CR were As (43.2%), Cr (29.1%), Cd (24.3%), and Pb (3.4%), emphasizing As’s toxicity and carcinogenic properties (highest CSF value of 1.5 mg/kg/day) even at low levels with no established safe intake limit. Processed cereals had the highest HI (3.45E-01, 67.5%) and ∑CR (1.09E-04, 62.5%) among food categories, followed by fish/meat products, beverages, milk/dairy products, and table salt. Although HI and ∑CR values were below thresholds, vulnerable groups (children, pregnant women) may face higher risks, and combined metal effects remain a concern.

**Table 4:** Hazard Quotient and Carcinogenic Risk of H.Ms from consumption of foodstuff samples.

Category	Sample type	Hazard Quotient (HQ)					Carcinogenic Risk (CR)				
		Pb	Cd	Cr	As	IH= ∑HQ	Pb	Cd	Cr	As	∑CR
Beverages	Carbonated soft drinks	6.36E-03	1.11E-02	3.05E-03	5.86E-03*	2.64E-02	1.89E-07	4.23E-06	4.57E-06	2.64E-06*	1.16E-05
	Energy drinks	7.32E-03	1.01E-02	4.37E-03	6.56E-03*	2.84E-02	2.18E-07	3.85E-06	6.56E-06	2.95E-06*	1.36E-05
	Tea	1.07E-03	1.71E-03	3.29E-04	1.90E-03	5.01E-03	3.19E-08	6.49E-07	4.94E-07	8.54E-07	2.03E-06
	Instant coffee	1.13E-04	2.25E-04	5.01E-05	2.50E-04	6.39E-04	3.35E-09	8.57E-08	7.51E-08	1.13E-07	2.77E-07
Processed Cereals	Wheat Flour	9.43E-02	2.91E-02	1.10E-02	6.47E-02	1.99E-01	2.81E-06	1.11E-05	1.65E-05	2.91E-05	5.95E-05
	Bread	1.77E-02	8.05E-03	1.26E-03	1.58E-02	4.28E-02	5.27E-07	3.06E-06	1.89E-06	7.1E-06	1.26E-05
	Macaroni	4.11E-03	1.76E-03	3.19E-04	2.13E-03	8.31E-03	1.22E-07	6.67E-07	4.79E-07	9.58E-07	2.23E-06
	Instant Noodles	1.41E-02	3.55E-03	1.39E-03	1.15E-02	3.05E-02	4.19E-07	1.35E-06	2.08E-06	5.18E-06	9.03E-06
	Corn products	1.12E-02	1.18E-02	2.15E-03	3.93E-02	6.45E-02	3.33E-07	4.48E-06	3.22E-06	1.77E-05	2.57E-05
Milk/Dairy products	Milk	5.33E-03	5.61E-03	1.57E-03	5.24E-03	1.78E-02	1.58E-07	2.13E-06	2.36E-06	2.36E-06	7.01E-06
	Dairy milk	5.65E-04	8.43E-04	2.22E-04	1.54E-03	3.17E-03	1.68E-08	3.2E-07	3.33E-07	6.94E-07	1.36E-06
Fish/Meat products	Canned Fishes	6.70E-03	1.08E-03	2.85E-04	3.04E-03	1.11E-02	1.99E-07	4.11E-07	4.27E-07	1.37E-06	2.4E-06
	Canned Beef	1.15E-02	1.77E-03	4.51E-04	4.86E-03	1.86E-02	3.43E-07	6.73E-07	6.77E-07	2.19E-06	3.88E-06
	Luncheon	1.57E-02	2.49E-02	6.56E-03	7.29E-03	5.45E-02	4.68E-07	9.46E-06	9.84E-06	3.28E-06	2.3E-05
Table salt	processed & natural	5.06E-04	8.40E-05	3.39E-05	2.63E-04	8.87E-04	1.51E-08	3.19E-08	5.09E-08	1.18E-07	2.16E-07

*The LOQ value of As was applied when undetected in the sample.

Links between dietary exposure and health outcomes (e.g., Pb and blood pressure, Cd and urinary cadmium) were based on international evidence (JECFA^30^; EFSA^16^ and included solely for contextualization of dietary exposure levels, as no biomonitoring was conducted in Egyptians. Although Pb poses relatively low carcinogenic risk, it has measurable cardiovascular effects. The results (Table 5) show that a daily intake of 6.88E-01 (µg/kg b.w. per day) of Pb from all studied food categories correlates with an estimated 5.29E-01 mmHg increase in systolic blood pressure. Although no defined global threshold exists for this increase, health guidelines recommend lowering Pb exposure to prevent hypertension. While there is a significant correlation between Cd and renal injury biomarkers, Table 5 shows that an intake of 1.12E-01 (µg/kg b.w. per day) of Cd from all food categories results in a UCd of 1.06E + 00 µg/g creatinine in adults, well below the JECFA safety limit of 5.24 µg/g creatinine^30^, indicating low renal risk from Cd exposure.

**Table 5:** The increase in systolic blood pressure due to Pb ingestion and the concentration of Cd in urine (UCd).

Category	Sample type	Blood pressure increase mmHg	UCd µg/g creatinine
Beverages	Carbonated soft drinks	1.71E-02	1.06E-01
	Energy drinks	1.97E-02	9.63E-02
	Tea	2.82E-03	1.58E-02
	Instant coffee	3.08E-04	2.22E-03
Processed Cereals	Wheat Flour	2.54E-01	2.77E-01
	Bread	4.77E-02	7.65E-02
	Macaroni	1.11E-02	1.67E-02
	Instant Noodles	3.79E-02	3.38E-02
	Corn products	3.01E-02	1.12E-01
Milk/Dairy products	Milk	1.43E-02	5.34E-02
	Dairy milk	1.52E-03	8.01E-03
Fish/Meat products	Canned Fishes	1.80E-02	1.03E-02
	Canned Beef	3.11E-02	1.68E-02
	Luncheon	4.24E-02	2.36E-01
Table salt	processed & natural	1.36E-03	7.98E-04
Total		**5.29E-01**	**1.06E + 00**

This study used deterministic estimates based on international reference values, inherent uncertainties related to exposure assessment and risk modelling. Without direct biomarker data or human validation, the results should be considered indicative rather than definitive. To enhance public health relevance, the findings of hazard and cancer risk should be contextualized and offered specific risk management recommendations. These include targeted monitoring of fish and meat products, especially luncheon meats, along with national regulatory H.Ms limits, and strengthening consumer awareness to minimize exposure. Incorporating such strategies can facilitate translation of scientific results into practical health protection measures. Lastly, while existing biomarker correlations provide a foundation for linking exposure to health outcomes, the lack of direct epidemiologicalor biomonitoring data from the studied population presents a limitation. Therefore, it is recommend future investigations integrate biomonitoring and health surveillance to validate modeled risks and improve exposure-response interpretations.

## Conclusion

Toxic heavy metals (Pb, Cd, Cr, As) in commonly consumed Egyptian foods and beverages were analyzed using Atomic Absorption Spectroscopy. The results highlight varying levels of heavy metals across food categories, with particular concern for products like canned fish, luncheon meats, and carbonated beverages. The metal content follows the order Pb > Cd > Cr > As, with the highest recorded values of Pb 298 µg/kg, Cd 134 µg/kg, Cr 99 µg/kg, and As 12 µg/kg in luncheon meat. While most levels adhered to established safe limits, Pb and Cd exceeded EFSA thresholds in luncheon meats, certain soft drinks, and dairy milk.

The potential health risk assessments for adults, based on estimated daily intake (EDI), Hazard Quotient (HQ), and carcinogenic risk (CR), suggest that exposure remains within health-based guidance values, ranking the dietary exposure as processed cereals > fish/meat products > beverages > milk/dairy products > table salt. HQ and CR values indicate minimal health risks overall, with minor effects of Pb intake on blood pressure and Cd exposure below safety thresholds.

However, it is important to note that these conclusions primarily reflect average exposures and do not fully account for vulnerable populations such as children, pregnant women, and individuals with higher consumption patterns or biological susceptibilities. Additionally, cumulative and aggregate exposures from multiple metals and dietary sources warrant further attention to better assess potential combined toxic effects and non-cancer health outcomes, which may pose a greater public health concern.

To address these concerns, we recommend specific risk management strategies including enhanced regulatory monitoring and control focused on high-risk food items (e.g., processed meats and canned fish), the implementation of routine heavy metal surveillance programs throughout the food supply chain, and public health education efforts to raise awareness about dietary heavy metal exposure and methods to minimize risks.

Moreover, greater emphasis should be placed on integrated risk assessments that incorporate synergistic interactions of multiple heavy metals and their chronic health impacts beyond carcinogenicity, alongside the inclusion of biomonitoring and epidemiological studies to validate and refine exposure-risk relationships for the Egyptian population.

Continuous assessment and improvement of food safety regulations, combined with these targeted strategies, are essential to effectively mitigate potential health risks associated with heavy metal exposure through diet and to protect vulnerable subgroups from adverse effects.

## Data Availability

All data generated or analyzed during this study are included in this article. The raw data supporting the conclusions of this article will be made available by the corresponding author, without undue reservation.
